# Assisting drinking with an affordable BCI-controlled wearable robot and electrical stimulation: a preliminary investigation

**DOI:** 10.1186/1743-0003-11-51

**Published:** 2014-04-07

**Authors:** Ritik Looned, Jacob Webb, Zheng Gang Xiao, Carlo Menon

**Affiliations:** 1 MENRVA Group, School of Engineering Science, Faculty of Applied Science, Simon Fraser University, 8888 University Drive, Burnaby, BC V5A 1S6, Canada

**Keywords:** BCI, EEG, Drinking task, FES, Stroke, Assistive, Exoskeleton, Hemiparetic, Upper extremity

## Abstract

**Background:**

The aim of the present study is to demonstrate, through tests with healthy volunteers, the feasibility of potentially assisting individuals with neurological disorders via a portable assistive technology for the upper extremities (UE). For this purpose the task of independently drinking a glass of water was selected, as it is one of the most basic and vital activities of the daily living that is unfortunately not achievable by individuals severely affected by stroke.

**Methods:**

To accomplish the aim of this study we introduce a wearable and portable system consisting of a novel lightweight Robotic Arm Orthosis (RAO), a Functional Electrical Stimulation (FES) system, and a simple wireless Brain-Computer Interface (BCI). This system is able to process electroencephalographic (EEG) signals and translate them into motions of the impaired arm. Five healthy volunteers participated in this study and were asked to simulate stroke patient symptoms with no voluntary control of their hand and arm. The setup was designed such as the volitional movements of the healthy volunteers’ UE did not interfere with the evaluation of the proposed assistive system. The drinking task was split into eleven phases of which seven were executed by detecting EEG-based signals through the BCI. The user was asked to imagine UE motion related to the specific phase of the task to be assisted. Once detected by the BCI the phase was initiated. Each phase was then terminated when the BCI detected the volunteers clenching their teeth.

**Results:**

The drinking task was completed by all five participants with an average time of 127 seconds with a standard deviation of 23 seconds. The incremental motions of elbow extension and elbow flexion were the primary limiting factors for completing this task faster. The BCI control along with the volitional motions also depended upon the users pace, hence the noticeable deviation from the average time.

**Conclusion:**

Through tests conducted with healthy volunteers, this study showed that our proposed system has the potential for successfully assisting individuals with neurological disorders and hemiparetic stroke to independently drink from a glass.

## Background

Much time and effort in recent years has been devoted to restoring function to paralyzed limbs resulting from hemiparetic stroke [1-3]. Traditional rehabilitative techniques require numerous sessions with a physiotherapist. These sessions are limited by the time and capabilities of the therapist; this in turn possibly limits the recovery of the patient [2]. Robotic aided rehabilitation [3-7] removes many of these limitations by performing the same rehabilitative and assistive motions accurately and without fatigue of the therapist. This potentially allows greater access to rehabilitative care for post stroke. An example is the ArmeoPower [8] a commercially available rehabilitative exercise device which provides intelligent arm support in a 3D workspace for individuals with neurological disorders.

Another popular method utilizing a different form of technology involves electrical stimulation of the user. Electrical stimulation of muscle groups has been used as both a purely rehabilitative technique to restore strength to atrophied muscles, and to manipulate the paralyzed limbs of both stroke patients and tetraplegics [9,10]. A voltage difference between the pairs of electrodes is generated which results in safe levels of current to flow through the region causing the activation of the respective muscle groups. A recent study [11] proposed using electrical therapy for conduction of tasks of daily living.

In addition to robot-aided rehabilitation and electrical stimulation therapy, brain computer interfaces have shown promising results in aiding stroke recovery [12-14]. Since the damage resulting from hemiparetic stroke is specifically limited to the brain itself, it is an intriguing solution to use a brain computer interface to help induce neuroplasticity [15]. Research has shown that simply imagining movement of a limb activates the same regions of the motor cortex as actually performing the movement [16]. Moreover, mental practice alone post stroke can help produce functional improvement [17].

Unfortunately each method alone has associated disadvantages, which prevent assisting activity of daily living and performing rehabilitation exercise at a comfortable setting such as the patient's home. Both rehabilitative and assistive robots are traditionally large and cumbersome which make them impractical to use outside of the laboratory environment [18]. The ArmeoPower being a prime example is not portable and is also currently not generally affordable by most of the patients [19]. In regards to FES, there are also different concerns, the primary one being fatigue in the respective muscle groups which may occur very quickly [20]. Similar setbacks with standard brain computer interfaces are they cannot be used outside of the laboratory environment due to their high cost and lengthy setup and training times.

Despite their disadvantages, each of the three technologies however shows peculiar promising aspects. Previous studies have in fact explored this concept and introduced combinational systems [21-28]. The work performed by Pfurtscheller et al. [29] is particularly relevant, as it investigated a BCI-controlled FES system use to restore hand grasp function in a tetraplegic volunteer. Another relevant study incorporating both BCI and FES focused on elbow extension and flexion [30]. A more thorough rehabilitative research comprised of a BCI system controlling a neuroprosthesis [31].

In this article, we propose a unique wearable and portable system that combines all three technologies for assisting functional movement of the upper limb that can potentially be used outside of the laboratory environment. Our proposed system consists of a wearable robotic arm orthosis (RAO) with functional electrical stimulation (FES), which is controlled through a BCI system. The RAO is an exoskeleton capable of providing active force assistance for elbow flexion/extension and forearm pronation/supination. The RAO is made of lightweight plastic with a compact design, and yet powerful enough to effectively assist the arm motion. The RAO does not assist shoulder motion due to the fact that 88% of stroke patients often have voluntary control over this region [32]. The FES is incorporated with the RAO to assist the hand in grasping/releasing an object. The use of FES is limited on the hand motion only, which allows reduction of fatigue and maintains the compactness of the system. Lastly, we seek to control the entire system using an affordable and portable BCI, which comprises of an inexpensive electroencephalography headset (Emotiv EEG headset) to acquire the brain signals and open-source software processing system, BCI2000. In order to evaluate the proposed system, a functional task of daily living - drinking a cup of water [33], is investigated. The drinking task consists of reaching for and grasping a cup from a table, taking a drink, and returning the cup to the table.

## Methods

### Robotic Arm Orthosis (RAO)

The goal was to design a system that was wearable and portable for enabling its future use in most activities of daily living (ADL). The robotic arm orthosis was developed to actuate the user’s elbow in flexion/extension as well as forearm pronation/supination. All structural components were fabricated out of an ABS derivative using rapid prototyping techniques.

**Figure 1:**
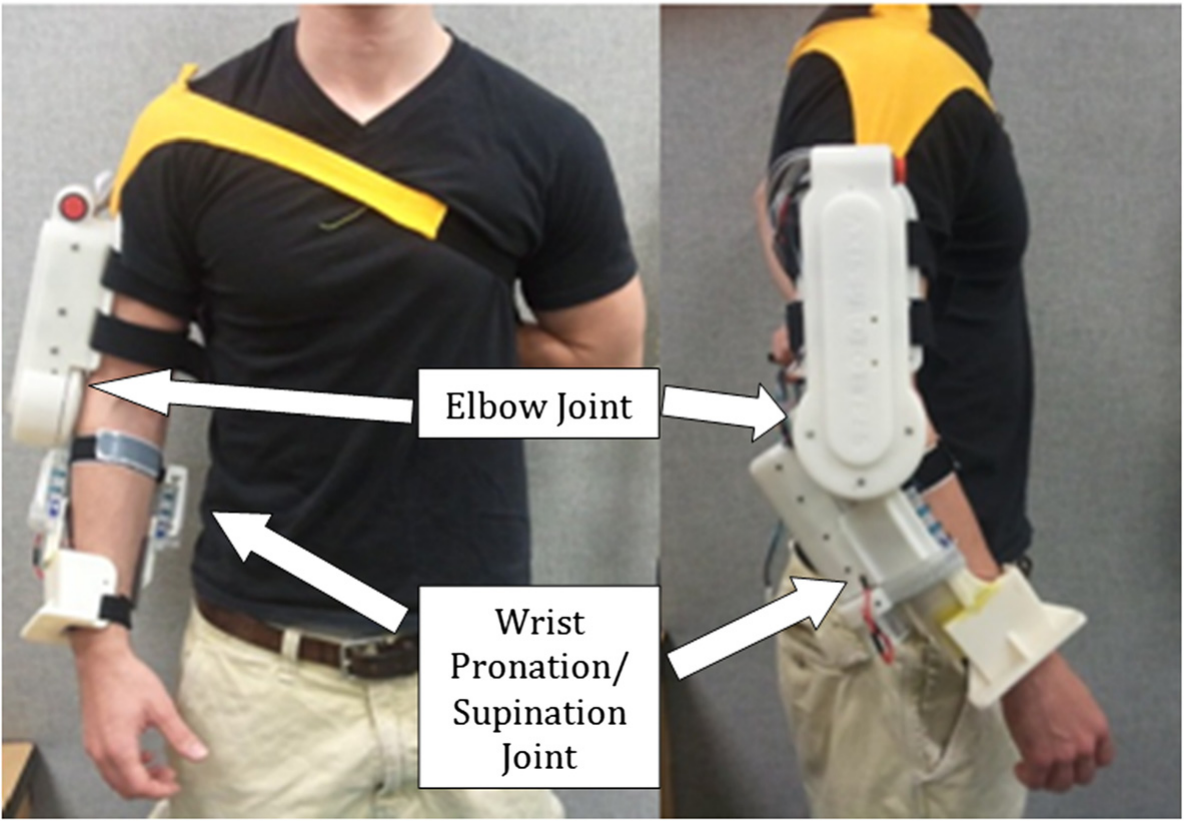
**Robotic arm exoskeleton.** Wearable, inexpensive device designed for assistive and rehabilitative purposes for stroke and spinal cord injury patients.

The elbow joint, as seen in Figure 1, was designed to generate 10 Nm of output torque by a brushless DC motor with customized gearbox, which is sufficient to lift the forearm if the user doesn't apply strong resistance force. Due to safety considerations the range of motion of the elbow assembly was mechanically limited to 110 degrees.

The pronation/supination joint of the ROA as seen in Figure 1, consists of two semi-cylindrical interlocking components. The upper component is fixed to the elbow joint while the lower component rotates freely within it and affixes to the user’s wrist. The lower component has a flexible chain wrapped around the outer surface that meshes with a pair of aluminum sprockets affixed to a motor and shaft assembly contained within the upper component. The system is capable of producing 75 degrees of rotation in both pronation and supination for a total of 150 degrees of movement. A brushed DC motor is coupled to a semi-cylindrical component to generate torque on the wrist.

As shown in Figure 1, the device is dawned on the user’s right arm as opposed to the left arm as most functional tasks are conducted with the right arm. Further, the feasibility scope of the study also granted us to assume that the right extremity was affected due to the stroke. The orthosis is affixed using two straps across the upper arm, one strap across proximal end of the forearm, one strap across the distal and of the forearm, and a final strap (yellow color in Figure 1) going over the user’s right shoulder and underneath the left arm. Donning the device takes less than 30 seconds when aided by another party and less than 60 seconds for an unaided healthy individual. Most of the weight is supported by the shoulder strap when the arm is relaxed at the user’s side. Both joints are positioned as such to not interfere with the user’s natural arm position whether relaxed or while performing tasks. The portability of the battery operated system allows the device to be used either as a rehabilitative aid in the laboratory, in the comfort of the patient’s home, or potentially as a functional device wherever the user may desire.

### Functional electrical stimulation

Stroke patients often have spasticity in their hands, which is an involuntary constant contraction of the muscles [34]. They are unable to voluntarily open their hands but are instead able to contract, as they desire. Therefore, hand opening was achieved by placing two electrodes on the distal and proximal ends of the extensor digitorum muscles of the forearm [35]. This provided the necessary contraction of the muscles to ensure the hand opened to a minimum degree required to grasp the cup.

For the purposes of this study an EMPI300 functional electrical stimulator from DJO Global was chosen in order to facilitate hand opening in patients who are otherwise incapable. The device itself is portable, battery operated, and capable of producing up to 50 volts at 100 mA. In addition, it is one of the few devices in which an external hand or foot switch may be attached to trigger stimulation. Extra circuitry was developed to interface the FES unit with the computer for control. When stimulation is externally triggered, the device can be set to ramp up to a predefined intensity within a preset period of time.

### Brain computer interface

In order to maintain the portability of the entire system, a wearable wireless EEG headset from EMOTIV was chosen to acquire data for the brain computer interface. The EMOTIV headset has 14 active electrodes operating at 2048 Hz before filtering. Amplification, buffering, and filtering are performed in the headset itself before being transmitted over a Bluetooth connection at 128 samples per second to a HP ENVY m6 laptop computer running an AMD A10 processer at 2.3 GHz with 8.0 GB of random-access memory (RAM). Hardware digital notch filters at 50 Hz and 60 Hz were utilized to filter out power line interference. Additional processing comprised of software filters, which include a spatial filter and a linear classifier. The objective of the spatial filter was to focus on the activity of the electrodes located over the sensorimotor cortex while the linear classifier was used to output actions corresponding to inputs.

For the purpose of this study we used the BCI2000 software, an open source system capable of data acquisition, stimulus presentation, and brain monitoring [36]. The four prominent modules accessible in this software include the source module (for data acquisition and storage), the signal processing unit, a user application, and the operator interface. BCI2000 is capable of utilizing the sensorimotor rhythms pattern to classify between motor movement and relaxation states. The sensorimotor rhythms were of significance in this project as alteration in the frequency and amplitude of these waves dictated actuation of the orthosis and FES. Sensorimotor rhythms consist of waves in the frequency range of 7 – 13 Hz (i.e. μ) and 13 – 30 Hz (i.e. β) and are evident in most adults typically around the primary sensorimotor cortices [37]. A decrease in the amplitude of the μ and/or β rhythm wave, known as Event Related Desyncronization [38], occurs upon both motor movement and imagined motor movement. Identifying this change then allowed us to classify the action as an imagined motor movement and activate the corresponding actuator.

One of the most prominent disadvantages of all BCI systems is the difficulty in differentiating between more than three classes in real-time sessions [39]. Classification accuracy in BCI systems decreases with the addition of classes. High-end BCI systems use complex algorithms and EEG caps with over 100 sensors in order to increase data resolution and therefore classification accuracy. This of course comes at the obvious expense of cost, setup time, and portability [40]. Therefore the highlight of our BCI is the ability to dynamically activate and deactivate pre-trained classes in real-time. This allows us to configure the system as to minimize the necessary classes. Thus, the system was designed to distinguish between only two cognitive classes (rest and motor imagery) and one artifact class (jaw clench).

**Figure 2:**
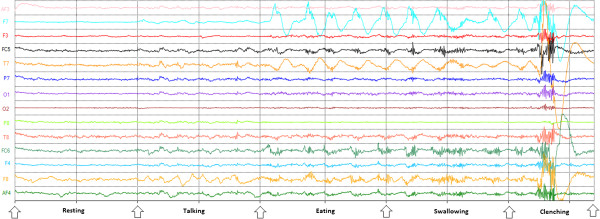
**Resting, Talking, Eating, and Swallowing vs. Clench.** Volunteers were asked to grind their rear molar teeth to switch between task phases. This produced a high frequency interference in the EEG signal making it a favourable distinguishable feature.

Since the jaw clench may interfere with other motions of the jaw during activities of the daily living, a preliminary test to assess the robustness of classifying this artifact was performed. Specifically, a test was designed to determine the accuracy of the clench artifact in which a volunteer was asked to alternate between talking, resting, eating, swallowing, and jaw clenching. The goal was to determine the accuracy of classifying clenching versus the other four jaw movements. A two-class problem was therefore formulated, in which one class was clenching and the other included talking, resting, eating and swallowing. Talking consisted of repeating an arbitrarily chosen phrase (i.e. "The quick brown fox jumped over the lazy dog", which includes a large number of letters of the alphabet); clenching of the jaw was performed by grinding firmly the rear molar teeth; resting was simply no talking and no jaw motions; eating consisted of chewing some food (a banana was arbitrarily selected); and swallowing entailed other gulping saliva or swallowing the chewed food. Figure 2 shows the EEG voltage-time signal visible during the five different motions considered in this test, namely resting, talking, eating, swallowing and clenching. In the performed test, a volunteer was asked to perform each of the five tasks for 10 seconds. The order of each task was randomized. Each 10 seconds phase was repeated 10 times for a total of 50 times. The entire procedure was performed five times. The average real-time classification accuracy performed with the inexpensive headset was of 98%. Results therefore suggest that jaw clenching can be robustly distinguished against other motions of the jaw typically performed in activities of daily living.

### Drinking task experiment

#### Experimental setup

**Figure 3:**
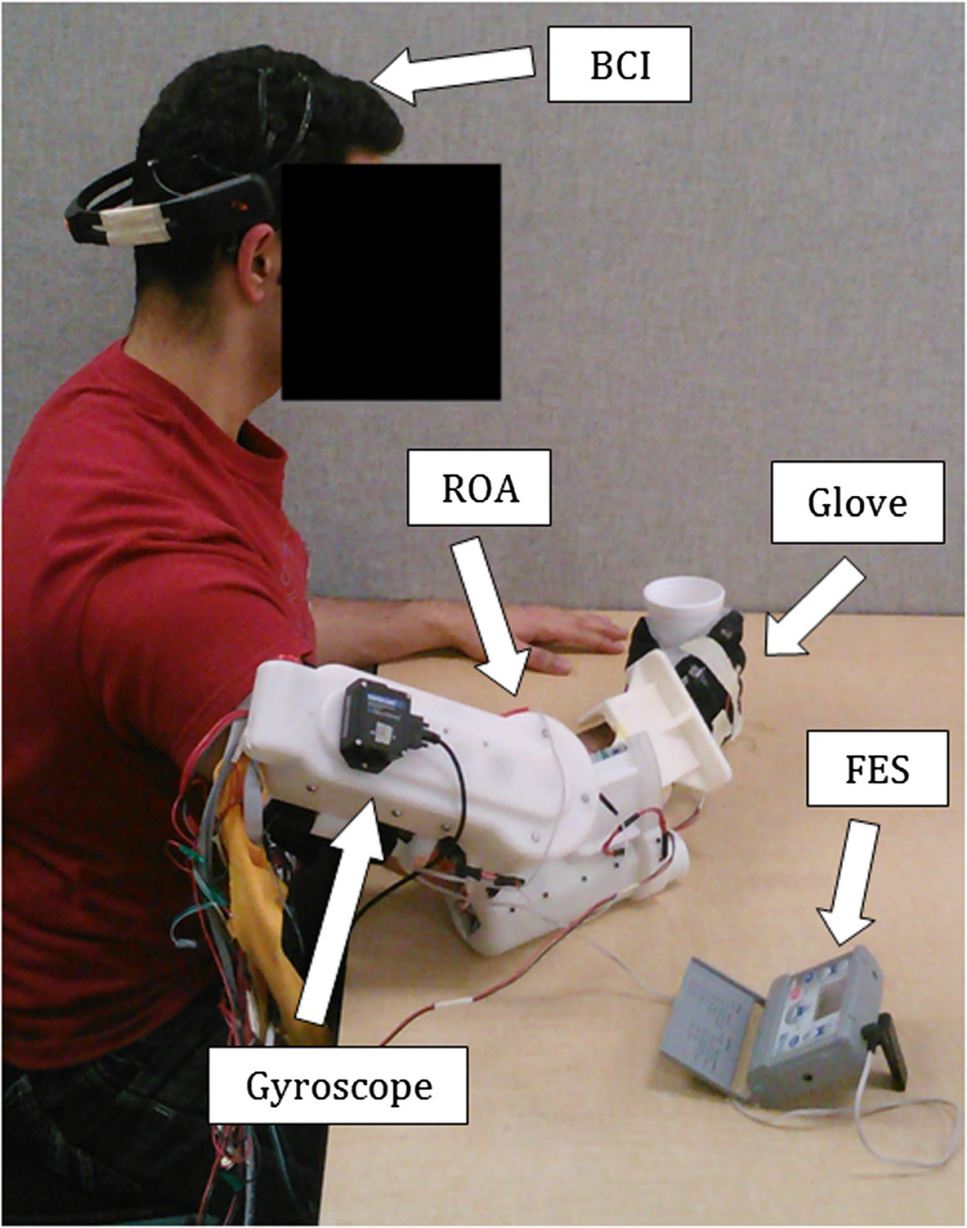
**Experimental Setup.** The complete system comprises of an EEG, FES, and a ROA. Additional measurement instruments include a custom glove worn on the right hand and a gyroscope attached to the upper arm.

As illustrated in Figure 3, the system setup required the user to wear the EEG headset on their head, the ROA on their arm, and the FES electrodes on their lower arm. An embedded potentiometer in the wrist pronation/supination joint provided measurable data on the wrist rotation angle while the encoder in the elbow motor was used to determine elbow angle. A custom designed glove with a fitted bend sensor along the middle finger was also worn by the individuals to track their hand configuration. A curled fist was calibrated to be 90° while a fully open hand was 0°. Data for measuring shoulder movement was recorded via a Microstrain Gyroscope affixed to the upper arm of the ROA. Vertical acceleration indicated motion of the arm as it was lifted up or brought back down to rest on the table.

#### Bci familiarization and demonstration

**Figure 4:**
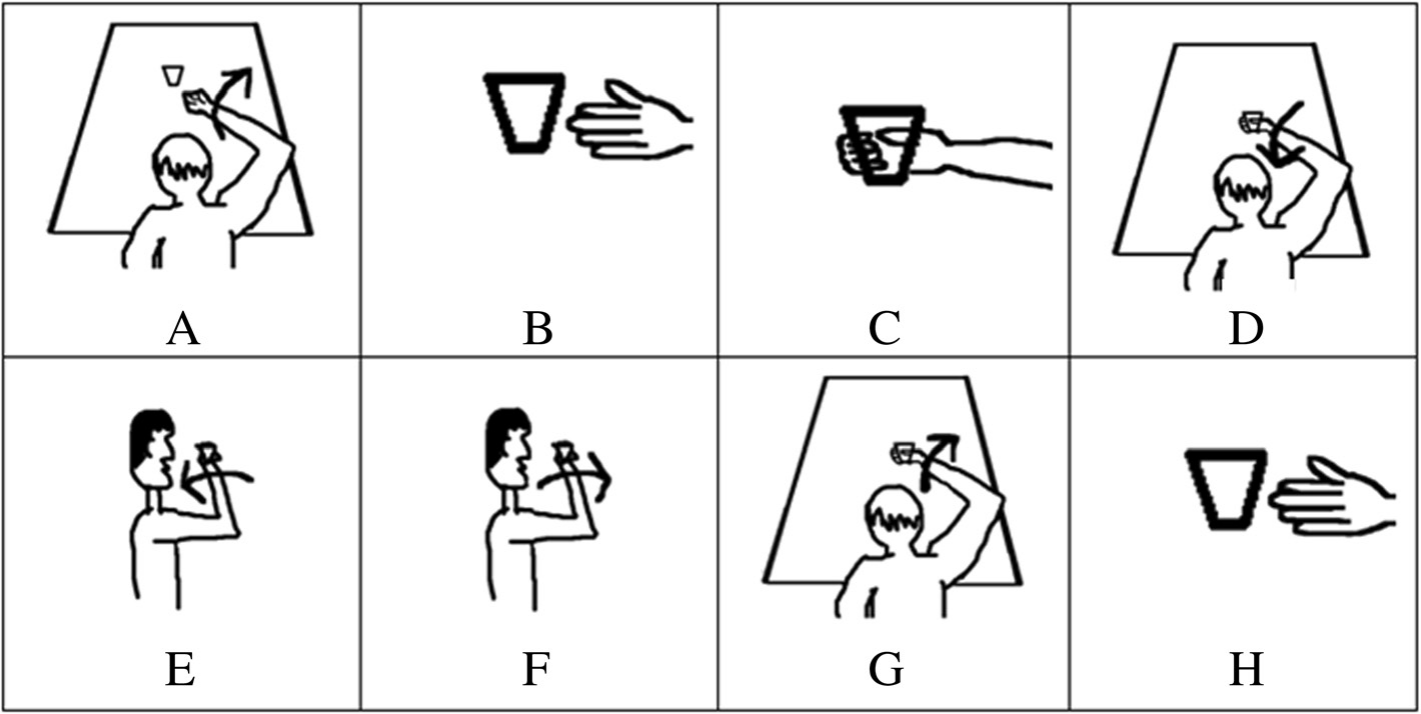
**Drinking Task Protocol.** Subjects were provided visual cues during the training phase to assist them in their formulation of their cognitive thoughts for each task. **(A)** Elbow extension. **(B)** Hand open. **(C)** Hand close. **(D)** Elbow flexion. **(E)** Supination. **(F)** Pronation **(G)** Elbow Extension. **(H)** Hand open.

**Figure 5:**
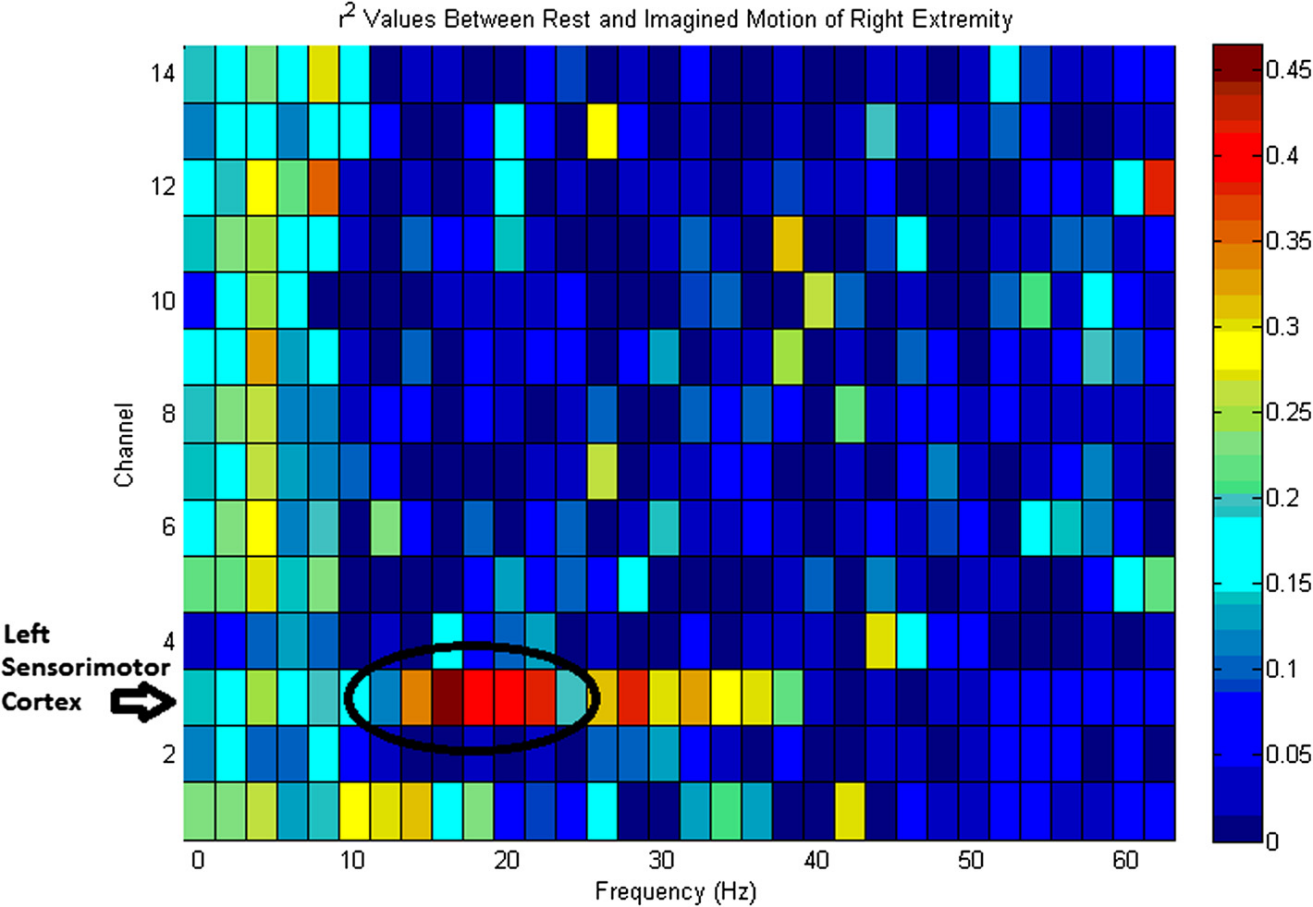
**BCI Feature Plot for rest vs. imagined motion.** The largest variation in the brain signal between rest and imagined motion occurs over the sensorimotor cortex. The electrodes corresponding to Channel 1–14 are AF3 F7 F3 FC5 T7 P7 O1 O2 P8 T8 FC6 F4 F8 AF4 respectively.

**Figure 6:**
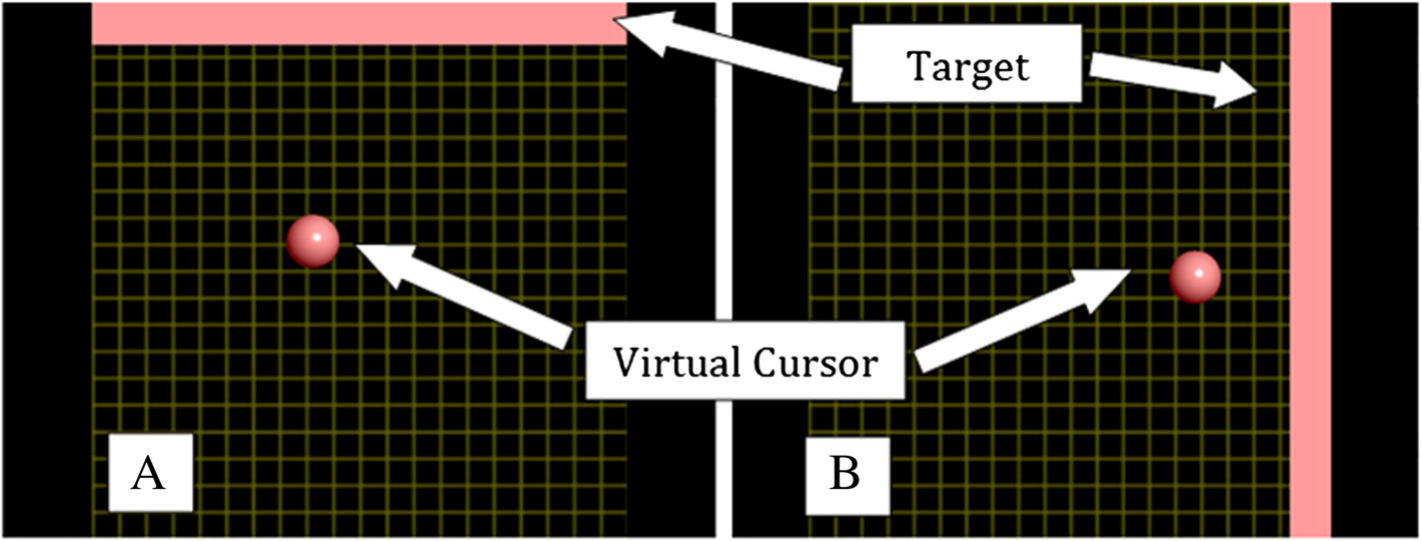
**User interface for the BCI system. (A)** Motor imagery thoughts translate the cursor in the vertical direction while **(B)** clenching of the teeth signals cursor translation in the horizontal direction.

Prior to experimentation all users were first introduced to the drinking task protocol which consists of the steps entailed in Figures 4A-4H. The trial consisted of an appearance of the Figures 4A through 4H in addition to a blank white screen in between transitions. Each image and blank screen was displayed for 5 seconds in which the volunteers were asked to imagine the corresponding motion for each displayed action and a neutral thought for the blank screen. This procedure was completed 2 times after which a feature plot (Figure 5) was generated. Based on the brain signal features, which differed the most between the two thoughts, parameters for the real-time task were configured. As expected, the most distinguishable features were in the mu and beta band over the left sensorimotor cortex. Following this initial phase, the volunteers were asked demonstrate their BCI control skill. The objective during the demonstration phase was to navigate the virtual cursor towards a virtual target as shown on the screen (see Figure 6). The target would either be visible at the top of the screen or to the right of the screen. Control of the cursor in the vertical direction towards the top target was induced by imagining an active thought, while facial clench controlled horizontal movement of the cursor towards the target on the right side of the screen. A neutral thought resulted in the virtual cursor to remain stationary.

A maximum duration of 5 seconds was provided to navigate the virtual cursor towards the target. The user was asked to demonstrate proficient BCI control (at least 80% accuracy) for each cognitive phase (elbow extension, hand open, elbow flexion, wrist pronation, wrist supination), before the subject was permitted to proceed onto the next segment of the experiment. In the case of extremely poor performance (accuracy less than 80%), another session at a later date would be conducted before the subject would be withdrawn from the study. This would be a rare possibility given that a similar field study conducted at an exposition in Austria with ninety-nine subjects indicated positive results [41].

#### FES intensity tuning

Following the BCI operation, the intensity of the FES system had to be tuned for each volunteer. Symmetrical biphasic pulses at a fixed frequency of 25 Hz were applied to all participants. This provided sufficient contraction of the digitorum muscles responsible for opening the hand. Studies have shown that higher frequencies (50 Hz) result in rapid fatigue [42] while low frequencies (15 Hz) are not adequate enough to recruit motor nerve units and only affect the sensory nerves [43]. The pulse width was also fixed at 200 μs for all subjects leaving the intensity parameter to be the only variable. The intensity of the stimulation was catered to each individual and was determined prior to initiating tests. The chosen values, as shown in Table 1, were programmed into the system and consequently applied during the experiment. A ramp up time of 1.0 second followed by continuous stimulation was initiated until the individual clenched their jaw to indicate termination of the task. No complaints of fatigue were expressed by the volunteers during any time of the experiment.

**Table 1:** Device intensity level and measured current across 1KΩ load at 25 V

	**EMPI intensity level**	**Current (mA)**
**Subject 1**	11	1.28
**Subject 2**	13	1.55
**Subject 3**	10	1.18
**Subject 4**	13	1.55
**Subject 5**	15	1.76

#### Drinking task protocol

**Figure 7:**
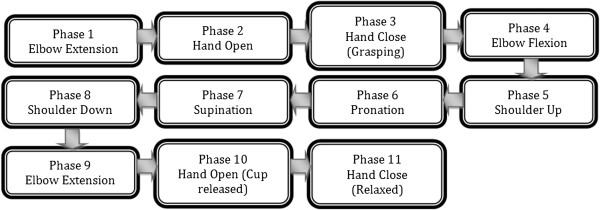
**Flowchart of Experiment.** The entire protocol consist of 11 phases, each operated sequentially. The phases are transitioned through by clenching of the jaw.

The protocol for the drinking task was split up into eleven sections (Figure 7), of which seven required the BCI and the rest consisted of voluntary movements. The voluntary movements were clenching of the jaw, hand contraction, and shoulder movement all of which can be performed by the majority of the stroke patients as discussed earlier. The transition from each section to the next required the subject to perform a clenching of the jaw. This artifact was used as it could be detected with high accuracy through the Emotiv EEG headset. Clenching of the jaw signified the end of the present phase and initiation of the next one.

Upon initialization of the drinking task, the elbow joint of the RAO automatically rotated to a “rest” position in which the user’s forearm was approximately horizontally parallel with the ground while resting on the table. The first motion of elbow extension (Figure 4A) was initiated by an imagined movement of the user extending their arm toward the cup. Once the thought was detected by the BCI, the elbow joint incrementally increased by a fixed angle, which was empirically selected to be 18° in this application. Repetition of the thought caused another incremental increase in the elbow joint. Once the volunteers were satisfied with their degree of elbow extension, they then clenched their jaw to move to the second phase. The second phase (Figure 4B) entailed the users’ opening of the hand by electrical stimulation. The electrical stimulation was initiated via an imagined hand open thought. A jaw clench turned off the FES which initiated the third phase of the task (Figure 4C). The user grasped the cup volitionally and the phase was completed once a finger flexion angle of 15° was detected. The angle of 15° was empirically selected. The fourth phase (Figure 4D) prompted the user to flex their elbow as to bring their arm towards their body. Increments of 18° were also used in this phase, which was terminated via a clenching of the jaw. The fifth phase was to bring the hand up towards the mouth. This required the user to utilize their shoulder voluntarily. Clenching of the jaw was used to indicate the end of the current phase. The sixth phase (Figure 4E) was wrist pronation, once again triggered by an imagined movement and terminated via a jaw clench. During this interval the user was expected to drink from the cup but this segment was neglected as to minimize the risk of any fluid spillage. Instead the users simply touched the cup to their lips. The seventh phase (Figure 4F) was wrist supination actuated in a similar manner but by imaging a supination motion. Each wrist rotation was empirically selected to be 60°. The eighth phase required the user to return their arm back on the table using their shoulder and clenching their jaw when done so. The ninth phase (Figure 4G) was once again elbow extension but this time with the cup in the hand. The corresponding imagined thought was the trigger mechanism. Incremental extension was terminated by a jaw clench. The tenth phase (Figure 4H) required the user to place the cup on the table by activating the FES unit. Once the users hand was opened, a clench terminated this phase and turned off the FES. The eleventh phase being the final phase simply required the user to voluntarily clench their hand. This motion indicated successful completion of the drinking task.

#### Participants

The goal of this study was to determine the capabilities of the complete RAO/FES/BCI system by performing a functional drinking task with healthy individuals simulating stroke patients. The users were asked to simulate the common spastic condition and allowed shoulder movements which parallel the abilities of stroke patients. The volunteers were also encouraged to not provide any volitional movement necessary for the task.

It should however be noted that possible undesired volitional movements of the participants could not compromise the validity of the performed tests. In fact, the RAO was not back drivable, and therefore did not allow movements of the elbow and pronation/supination without a correct use of the BCI interface. In addition, electromyographic (EMG) signals were also used to monitor activities of the different muscles of the participates’ hand to make sure they did not interfere during the phase in which FES was used to clench their hand.

Five healthy individuals (mean age equal to 21 ± 1 yr) with no prior experience with EEG-based BCI systems volunteered to participate in the research (project approved by the Office of Research Ethics, Simon Fraser University).

## Results and discussion

### Drinking task results

**Figure 8:**
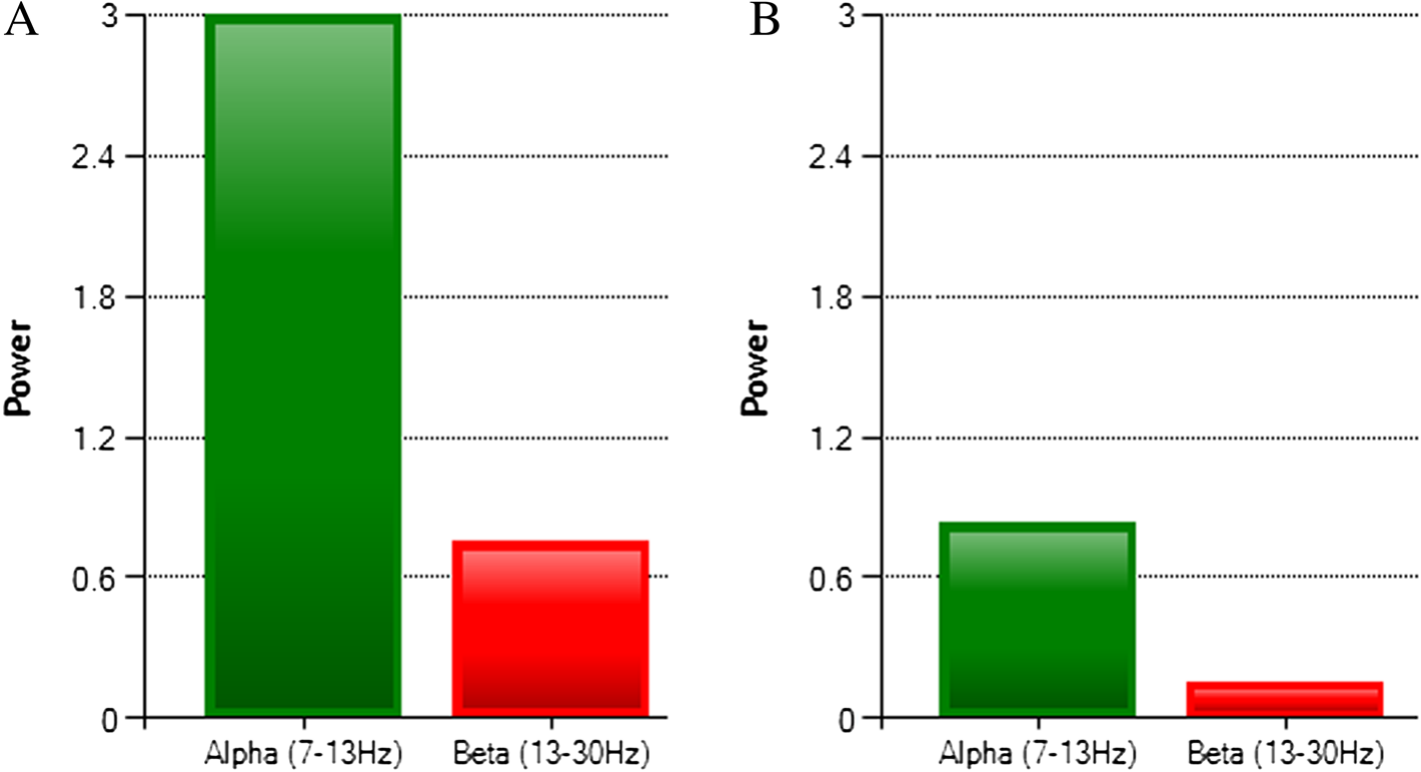
**Real-time screenshot of Event-Related-Desynchronization.** Power spectrum at rest **(A)** compared to the power spectrum just prior to imagined motion **(B)** of the upper extremity during the drinking task. Suppression of the mu and beta frequency bands is the trigger signal for each actuator.

**Figure 9:**
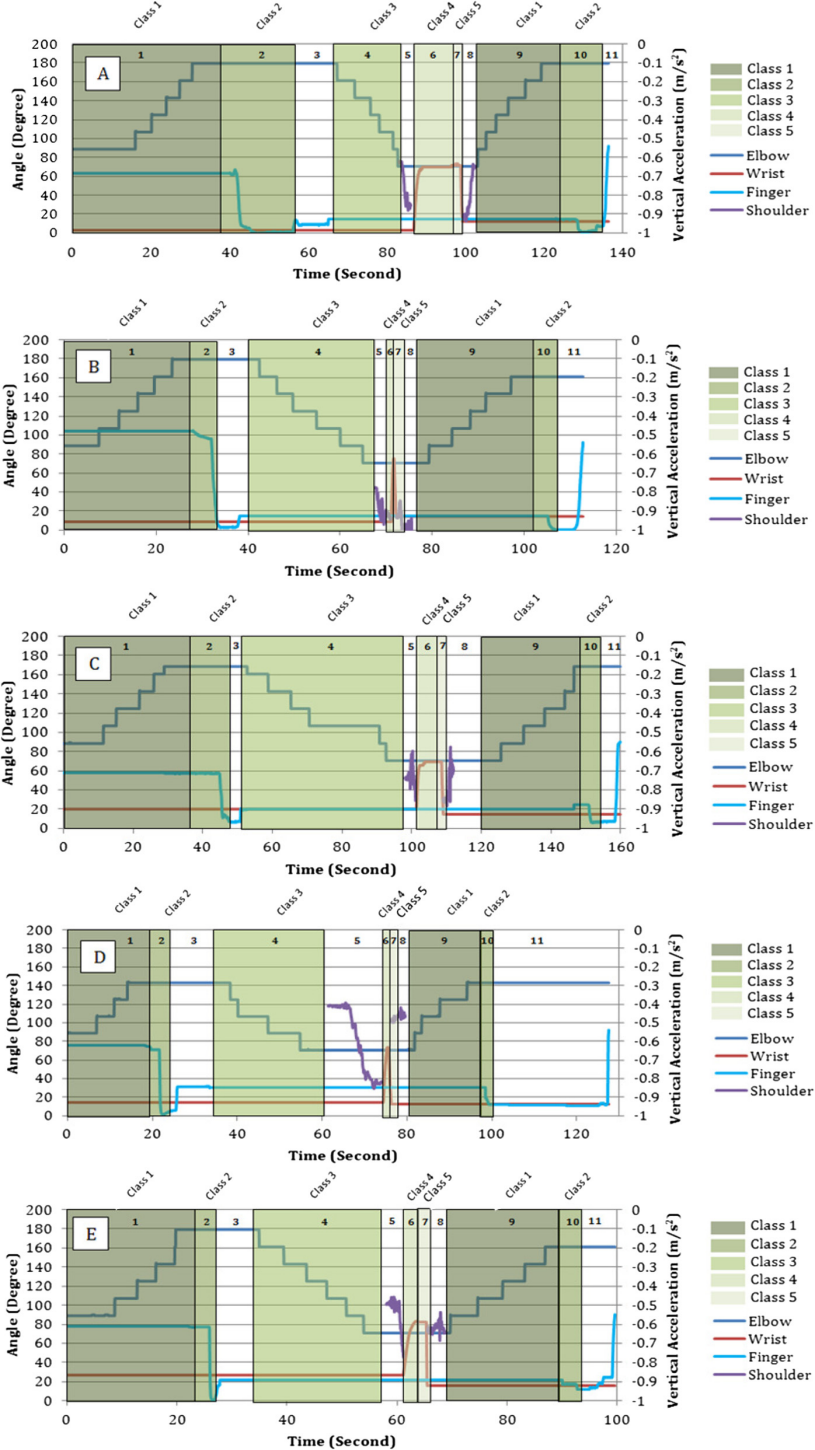
**Presentation of extremity throughout the drinking task.** Elbow movements are incremental. Finger flexion and wrist rotation are continuous. Shoulder acceleration is only during motion. Class 1 – Elbow Extension. Class 2 – Elbow Flexion. Class 3 – Hand Open. Class 4 – Pronation. Class 5 – Supination. Unshaded – Volitional. **(A)** Subject 1. **(B)** Subject 2. **(C)** Subject 3. **(D)** Subject 4. **(E)** Subject 5. Each phase of the task is labeled in the respective regions.

Once the volunteers demonstrated competency in controlling their μ rhythm brain activity, they were then asked to perform a complete drinking task motion. A snapshot of the real-time data captured over the sensorimotor cortex during the elbow extension phase of the drinking task illustrates the ERD effect (Figure 8). A decrease in the amplitude of the mu band and the beta band was visible prior to each motion for all subjects. Rather than specifying a constant numerical threshold for the amplitude, the system computed the average EEG power decrease relative to a reference value over fixed time intervals. This approach compensated for each individual and eliminated the need for any manual adjustments. Data from the elbow joint, middle finger joint, wrist, and shoulder were all recorded during their respective active phases and are illustrated in Figures 9A – 9E. Each BCI phase of the task is indicated with a different shade of green while the un-shaded parts are the volitional movements. Additional numbers are labeled to indicate the exact phase sequences which were defined earlier.

As indicated in Figures 9A – 9E, elbow extension and flexion for all users occurs in increments. The incremental method was chosen to provide users with a controllable range of motion as opposed to a full continuous extension or flexion. This was necessary as seen by the results of the user in Figure 9D. This user did not fully extend the arm as opposed to other individuals yet was still able to grasp the cup and fully complete the task. Full flexion of the elbow (71°) on the contrary was necessary for all individuals as to ensure that the cup would be able to make contact with the lips.

Wrist pronation and supination on the other hand occurred in a single smooth motion with a rotation of approximately 70°. This provided the necessary tilt to allow drinking from the cup. Although users were not drinking in these trials due to safety concerns mentioned earlier, the simulation imitated the action reasonably.

Analysis of the results provided in Figures 9A – 9E further indicate that each user performed the task at their own pace. For example, comparison of the elbow extension phase for Figure 9A and Figure 9B illustrates the variance in BCI control. Although both users fully extended their elbow during this phase, one user took 40 seconds while the other user took 28 seconds (see Table 2). Additional differences in the time taken to complete each phase vary throughout the entire protocol for each volunteer. This applies to both the BCI controlled motions and the volitional motions. As a consequence, the total time for completion varied with the individual.

**Table 2:** Duration to complete each phase of the task and to complete the entire protocol

	**Arm extension**	**Hand open**	**Arm flexion**	**Pronation**	**Supination**	**Arm extension (2)**	**Hand open (2)**	**Total**
**Subject 1**	39.9 s	15.6 s	17.8 s	12.7 s	0.55 s	20.5 s	11.3 s	136.4 s
**Subject 2**	27.9 s	7.6 s	29.4 s	0.58 s	0.52 s	25.1 s	8.75 s	112.8 s
**Subject 3**	28.9 s	18.8 s	45.6 s	7.2 s	0.56 s	34.5 s	11.4 s	159.9 s
**Subject 4**	17.5 s	5.3 s	27.8 s	0.6 s	1.0 s	18.9 s	6.4 s	127.5 s
**Subject 5**	22.0 s	4.5 s	25.9 s	2.3 s	0.62 s	21.3 s	3.5 s	99.6 s

As stated earlier, the time required to complete the whole drinking motion depended upon how well the subject was able to control their cognitive thoughts and at what pace they conducted their voluntary movements. Overall, the duration ranged from 100 seconds to 160 seconds for the individuals to complete the task. The average time to complete the motion was 127 seconds with a standard deviation of 23 seconds.

The results attained further indicate the practicality of the system in terms of the duration it takes to complete a drinking task. The values are fair and can be analyzed over a period of trials to visualize improvements in the patients’ abilities when testing with stroke or spinal cord injury patients.

### Limitations of study

Although optimistic results were presented in this study, some assumptions were made during the project design. Firstly, determining the true intention of the users when operating the BCI was based on the users’ word. In fact, to the best of the authors’ knowledge, there is no verification method to establish that the user was indeed imagining ‘reach’ during the elbow extension phase and not imagining a different motion. It is understandable to assume, as we did, that the users will follow the guidelines when using the system for both an intuitive experience and maximal benefit for themselves.

Secondly, while the study is in fact designed for stroke and spinal cord injury patients, it currently lacks that portion. The next step would be to test the system with individuals who would actually benefit from the operation.

**Figure 10:**
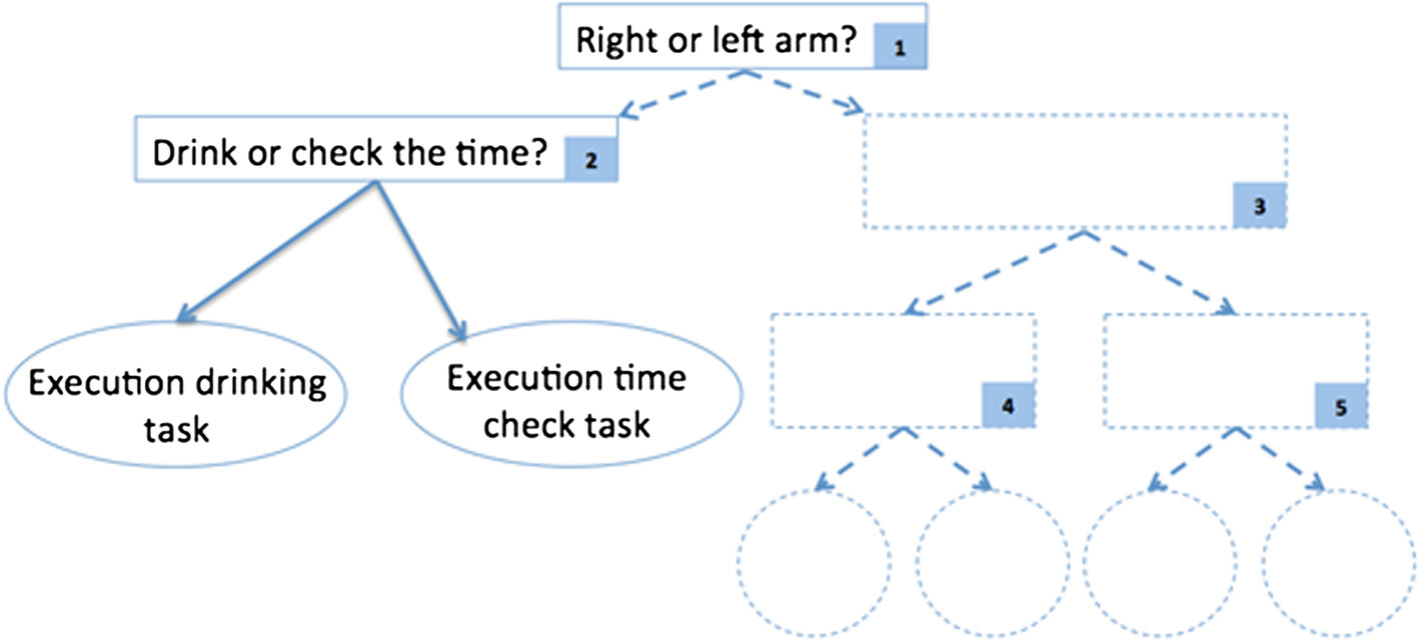
**Cascaded classification scheme.** Example strategy to potentially achieve assistance in multiple functional tasks of the upper extremities.

Lastly, the current study was limited to the sole functional task of drinking a glass of water, as the main focus was to highlight the feasibility of such a combinational system of an ROA, FES, and BCI for a single functional task of the arm. Future versions, while maintaining the inexpensive and portable nature of the setup, may include other functional tasks. It should be noted that the ROA and FES could already potentially assist a large variety of tasks in their current configurations. In fact, they could assist a number of functional tasks including reaching for an object, turning the handle of a door, supinating the arm to see the time on a wrist watch, pulling a drawer, and grasping an object to move it from one location to another. The main challenge to obtain a multi-task system is therefore the identification of the user’s intention using the BCI. A potential approach is the use of a cascaded classification strategy [44]. In fact, similarly to a decision tree [45], a binary classifier could be used in each internal node of the tree. This approach would enable selecting a number of tasks, corresponding to the number of the leaves of the tree. An example of feasible binary classification consists of determining if the volunteer intends to move her/his right or left extremities – such a classification was proven to yield high accuracy [46-49]. This node would be of interest in case the volunteer wears assistive devices on both arms. Another set of tasks for a potential node would be the “drinking task”, as analyzed in this manuscript, and another functional task, such as a “time check task”, consisting of supinating the forearm to check the time on a wristwatch. The functional mental task used to identify the intention of the user to drink from a glass of water would be to imagine being thirsty and wanting to reach out for a glass placed in front of the volunteer. Initiation of the time check task would instead be triggered by imagining a ticking clock where the user is asked to continuously add the numbers on the dials. This two-task problem has strong potential for being classified with high accuracy as research showed that motor and arithmetic tasks could clearly be distinguished [50-52]. An example of the suggested cascaded classification to be investigated in future research is presented in Figure 10. In addition to the above-mentioned cascaded approach, the advancement of BCI systems could also enable the future extension of the proposed two-class problem to multi-class problems. Research has shown strong potential in this regard. For instance, Schlögl et al. demonstrated the feasibility of classifying four motor imaginary tasks [53]. Future research aimed at on-line classification of motor tasks using inexpensive headsets could therefore further facilitate the use of the proposed technology for assisting multiple tasks.

## Conclusion

The goal of this study was to explore an inexpensive and fully portable assistive technology for individuals with neurological disorders in their pursuit of independently drinking from a glass. A combination of a robotic arm orthotic, an electrical stimulation system, and a Brain Computer system were utilized for this task. The task consisted of a drinking maneuver broken down into eleven phases where each phase was triggered by the respective imagined movement and terminated by a soft clench of the jaw. The ambitions of the study were met with five healthy volunteers who simulated stroke patients with spasticity. The volunteers completed the drinking maneuver with an average time of 127 seconds and a standard deviation of 23 seconds. The next step would be expand the capabilities of the system by including additional functional tasks and then conducting tests with stroke and spinal cord injury patients to assess the benefit of the system.

## Competing interests

The authors declare that they have no competing interests.

## Authors’ contributions

CM conceived the work, proposed the overall protocol, and directed the development of the robotic system. ZGX assisted on selecting the components of the system, programming the robot and interpreting the recorded data. JB designed the robot, interfaced the Emotiv EEG headset to the robot, and contributed to the design of the protocol. RL interfaced the Emotiv EEG headset to the BCI2000 software and to the FES, conducted the tests with the volunteers, and finalized the protocol. All authors were involved on drafting the paper and read and approved the final manuscript.
